# Do diet and Fumagillin treatment impact *Vairimorpha* (*Nosema*) spp. (Microspora: Nosematidae) infections in honey bees (Hymenoptera: Apidae) and improve survival and growth of colonies overwintered in cold storage?

**DOI:** 10.1093/jee/toae187

**Published:** 2024-09-28

**Authors:** Gloria DeGrandi-Hoffman, Vanessa Corby-Harris, Henry Graham, Mona Chambers, Emily Watkins deJong, Lucy Snyder

**Affiliations:** USDA-ARS, Carl Hayden Bee Research Center, 2000 East Allen Road, Tucson, AZ, USA; USDA-ARS, Carl Hayden Bee Research Center, 2000 East Allen Road, Tucson, AZ, USA; USDA-ARS, Carl Hayden Bee Research Center, 2000 East Allen Road, Tucson, AZ, USA; USDA-ARS, Carl Hayden Bee Research Center, 2000 East Allen Road, Tucson, AZ, USA; USDA-ARS, Carl Hayden Bee Research Center, 2000 East Allen Road, Tucson, AZ, USA; USDA-ARS, Carl Hayden Bee Research Center, 2000 East Allen Road, Tucson, AZ, USA

**Keywords:** overwintering, cold storage, colony population dynamics, pollen, protein supplement

## Abstract

*Vairimorpha* (Microsporidia: Nosematidae) is a microsporidian that infects honey bees especially in winter. Fumagillin can reduce infections, but whether overwintering survival is improved is unclear. The diet also may influence the severity of *Nosema* infections. We examined the relationship between *Nosema* and colony size and survival in hives overwintered in cold storage facilities. In year 1, no Fumagillin treatments were applied. Colony size and survival after cold storage and almond bloom were comparable between groups with high and low pre-cold storage infections. In year 2, size and survival were compared among colonies with and without Fumagillin treatment that were fed either pollen or protein supplement prior to overwintering. Colonies treated with Fumagillin had lower spore numbers than untreated, but colony sizes and survival were similar among the treatments. However, more colonies with zero spores per bee could be rented for almond pollination and were alive after bloom than those averaging >1 million spores per bee. Fat body metrics can affect overwintering success. In both years, fat body weights and protein concentrations increased, and lipid concentrations decreased while bees were in cold storage. Fat body metrics did not differ with *Nosema* infection levels. However, Fumagillin negatively affected pre-cold storage fat body protein concentrations and colony sizes after cold storage and almond bloom. Treating with Fumagillin before overwintering in cold storage might result in greater colony survival if spore numbers are high, but undetectable or even negative effects when spore numbers are low.

## Introduction

The health of honey bee colonies can be compromised by various stress factors, none more important than the incidence of disease. Honey bees are hosts to numerous pathogens, many of which are acquired by foragers on floral resources (McArt et al. 2014, Koch et al. 2017, Dalmon et al. 2021, Van Wyk et al. 2023). One pathogen that can be picked up from flowers via a fecal–oral route is the microsporidian parasite, *Vairimorpha* (*Nosema* spp.) (Graystock et al. 2015, Tokarev et al. 2020). Historically, honey bees were infected with *Nosema apis*. In recent years, *N. ceranae* infections are more common suggesting that it is replacing *N*. *apis* in many areas (Chen et al. 2008, Botías et al. 2012, Martín-Hernández et al. 2018).

All ages of worker bees can become infected with *Nosema*. Worker bees infected as larvae have poorly developed brood food (hypopharyngeal) glands (Alaux et al. 2010, Jack et al. 2016, DeGrandi-Hoffman et al. 2018), transition to foragers earlier in their adult life (i.e., precocious foragers) (Goblirsch et al. 2013, Natsopoulou et al. 2016), and have shorter lifespans compared with uninfected bees (Mayack and Naug 2009, Eiri et al. 2015, Urbieta-Magro et al. 2019). Infected bees also show symptoms of energetic stress and apparent hunger (Mayack and Naug 2009), perhaps because *Nosema* appropriates nutrients from host cells (Paris et al. 2018). During long periods of confinement in the hive as in fall and winter in temperate areas, spore numbers in bees can increase rapidly.

Though the negative impacts of *Nosema* infections on individual bees are well documented, the effects on colony growth and survival are less consistent. *Nosema* can be highly pathogenic to honey bees and reduce brood rearing and honey production leading to the eventual collapse of colonies (Martín-Hernández et al. 2007, Higes et al. 2009, Botías et al. 2013). Other studies, however, do not support a relationship between *Nosema* and colony failure (Genersch et al., 2010, Gisder et al. 2010, Dainat et al. 2012) or show that other causes such as Varroa mites (*Varroa destructor* Anderson & Trueman) and the viruses they transmit are largely responsible for colony loss (Schüler et al. 2023). Some of the discrepancies among studies could be from differences in the severity of *Nosema* infection. A recent study reported that *Nosema* infections are negatively correlated with colony size when spore levels are greater than 1 million spores per bee (Emsen et al. 2020). Colonies with highly infected short-lived workers will fail to grow and eventually collapse because brood rearing is connected to adult population size. Concurrent declines in adult bees and brood can drive populations below a critical threshold where they cannot recover (DeGrandi-Hoffman and Curry 2004, Betti et al. 2014, Dennis and Kemp 2016, Booton et al. 2017, Petric et al. 2017, Chen et al. 2021). Colonies are particularly vulnerable to collapse if reduced longevity occurs in the fall when brood rearing is declining or in the spring when foraging resumes and brood rearing is needed to replace older foragers (Betti et al. 2016).

Virulence and colony loss from *Nosema* may be mitigated by management practices. Nutrition seems to play a role as the type of food bees consume can impact the growth rate of spores and the longevity and survival of infected bees. Infected colonies on natural forage or fed pollen have lower spore numbers than those fed protein supplements (Fleming et al. 2015, DeGrandi-Hoffman et al. 2016, Watkins de Jong et al. 2019). In cage studies, though, the prevalence of *N. ceranae* increased in bees-fed diets with higher pollen quantities, but there also was greater bee survival and longevity (Jack et al. 2016).

Another management option to control *Nosema* is treating bees with mycotoxin, Fumagillin (dicyclohexylamine salt) (Higes et al. 2011). Fumagillin is the only registered compound for treating *Nosema*. However, overuse resulting in resistance is a concern, as are Fumagillin residues persisting in hives and contaminating honey (Lopez et al. 2008, van den Heever et al. 2016). There also can be toxic side effects from Fumagillin, such as reduced brood rearing (Webster 1994), alteration of structural and metabolic proteins in the midgut (Huang et al. 2013), and reduction in sperm quality (Abdelkader et al. 2021). If management practices could reduce the proliferation or impact of *Nosema* in colonies, Fumagillin applications may not be needed as frequently, reducing the chances of resistance and lowering the costs involved in treating this pathogen.

To determine if management practices could reduce colony losses from *Nosema*, we conducted a study with commercial honey bee colonies that were overwintered in cold storage and used for almond pollination. We chose to overwinter colonies in cold storage because of lower colony mortality than those overwintered outdoors (Williams et al. 2010, Punko et al. 2021, Hopkins et al. 2023) and assurance that all treatment groups would be exposed to the same overwintering conditions. In year 1, colonies were not treated for *Nosema* prior to cold storage. Colony sizes after cold storage and almond bloom were compared between those with high and low *Nosema* infections prior to cold storage. In year 2, the study was expanded to incorporate the effects of diet and Fumagillin treatments on pre-cold storage *Nosema* infections and colony sizes after cold storage and almond bloom. Because the fat body is a key storage organ while bees are confined in the hive and *Nosema* can affect nutrient acquisition (Aliferis et al. 2012), we measured fat body metrics before and after cold storage to determine if they were affected by *Nosema* infection levels or Fumagillin treatment. Finally, an economic analysis was conducted to determine the profitability of Fumagillin treatments prior to cold storage based on costs for the treatment and the difference in the percentage of treated and untreated colonies that could be rented for pollination.

## Materials and Methods

### Overview of the Study

**Fig. 1.**
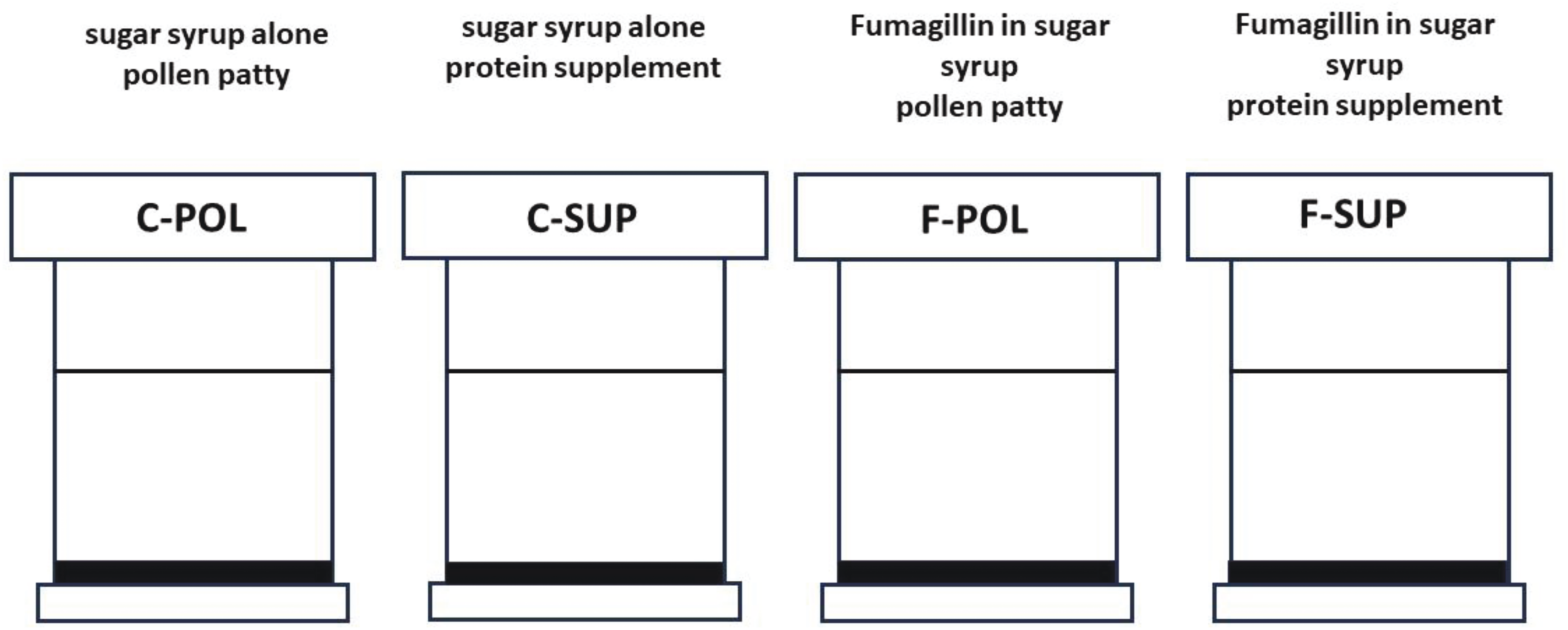
Treatment groups established in year 2 to evaluate the effects of diet and Fumidil-B (Fumagillin) treatment to control *Nosema spp*. infections. Colonies were fed either pollen (POL) or protein supplement (SUP). Fumidil-B was applied by feeding it to colonies in sugar syrup according to the manufacturer’s instructions (F-POL and F-SUP). Control colonies (C-POL and C-SUP) were fed sugar syrup without Fumidil-B. *Nosema* spores per bee were measured before and 3 weeks after diet and Fumagillin treatments.

In both years of the study, we used commercial honey bee colonies headed by European queens and summered in Baldwin, ND, USA (47° 1ʹ35.2920″ N 100° 44ʹ 57.9408″ W). Colonies were selected based on population size (>8 combs with bees and brood) and low mite numbers (<1 Varroa mite per 100 bees) in September. In year 1, 39 colonies were selected to evaluate the effects that *Nosema* levels prior to cold storage overwintering had on post-cold storage and post-almond bloom colony size. In year 2, we expanded the study to 352 colonies and included the effects of diet and Fumagillin treatments on *Nosema* spore numbers and subsequent colony size and survival after overwintering in cold storage and pollinating almonds. Colonies were randomly assigned to one of 4 treatment groups (88 colonies per group) and sampled (pretreatment) for *Nosema*. pretreatment averages of spores per bee did not differ among treatment groups. Colonies were fed either pollen (POL) or the protein supplement (SUP) Global Patties (Global Patties, Airdrie, AB, Canada), which contain 4% pollen. Pollen was fed as patties that contained 50% dry pollen by weight (polyfloral mixture), 25% granulated sugar, 24.5% water, and 0.5% citric acid as a preservative. Colonies fed either pollen patty or protein supplement were treated with Fumagillin administered as Fumidil-B in sugar syrup (F-POL and F-SUP, respectively) according to manufacturer’s directions (KBNP, Chungnam, Korea). Control colonies were fed pollen (C-POL) or protein supplement (C-SUP) and sugar syrup only (Fig. 1). In year-1, colony sizes were measured in the last week of September just prior to cold storage (pre-cold storage evaluation), and again after cold storage (post-cold storage) and almond bloom (post-almond bloom). In year-2, colony sizes were measured before (pretreatment) and 3 weeks after diet and Fumagillin treatment (pre-cold storage), after cold storage overwintering (post-cold storage) and after almond bloom (post-almond bloom).

Samples of worker bees (20 per colony) were taken from colonies in year 1 to estimate *Nosema* spores prior to cold storage (7 October 2019) (pre-cold storage). In year 2, samples were taken on 28 August—1 September 2021 to determine *Nosema* levels (pretreatment sample). Three weeks after diet and Fumagillin treatments (25–26 September), sampling was repeated (pre-cold storage). In both years, samples of bees for *Nosema* were taken from honey frames. Samples of bees for fat body analysis were taken from brood frames. All samples (*Nosema* and fat body) were packed in dry ice, shipped to the USDA-ARS, Tucson, AZ, USA, and stored at −80 °C until analysis.

Hives were moved to a cold storage facility for overwintering (Bee Storages of Idaho LLC., Filer, ID, USA) during the first week in October in both years of the study. Hives remained in the facility until the last week in January, when they were shipped to California almond orchards. Colonies were evaluated during the first week in February for combs with bees and brood (post-cold storage colony size). Colonies also were sampled for fat body analysis using methods described above (post-cold storage fat body metrics). Since the bees were able to fly and defecate for several days before our measurements, we did not sample for *Nosema* in either year of the study because spore numbers might have under-represented infection levels in bees while confined in the hive. After almond bloom, colony sizes were measured a final time (post-almond bloom colony size).

### Estimating Colony Size

In years 1 and 2, combs of adult bees and sealed and unsealed broods were measured on Langstroth deep frames (comb dimensions: 48.3 cm × 2.7 cm × 23.2 cm). Bees and brood were measured using methods adapted from Delaplane et al. (2013). Bees in sections representing one-tenth of the comb area were counted on both sides of each frame and summed to estimate combs of bees and brood.

In both years of the study, temperatures were below 10 °C during the pre-cold storage colony measurements, prohibiting the use of the above-mentioned method of comb evaluations. Instead, adult populations were measured using the California almond pollination method (Goodrich and Goodhue 2016). This method describes a Langstroth deep frame 75% covered in adult bees as a frame or comb of bees. Colonies in each treatment group consisted of 2 deep Langstroth hive boxes. To estimate colony size, the hive bodies were tilted forward on the pallet and frames with at least 75% bee coverage were counted. In the lower hive body, frames were counted from the top, and in the upper hive body, frames were counted from the bottom. Frames with bees were totaled for the colony as an estimate of colony size.

### Estimating Mite Populations

Samples of adult workers were taken on 6–12 September 2019 (year 1) and 28 August–1 September 2020 (year 2) to estimate Varroa infestations. Approximately 300 workers per colony were sampled by brushing bees from brood comb into jars containing 50 ml of 70% ethanol (Dietemann et al. 2013). Samples were refrigerated until analyzed. Mites were counted by vigorously shaking the sample vials for 20 s, and then pouring the bees and alcohol into a strainer positioned over a pan. The mites that went through the strainer and into the pan were counted. We also examined the bees in the strainer for mites that remained on the bees. All the bees in the sample were counted to estimate mites per 100 bees (Dietemann et al. 2013). Colonies were treated for Varroa with 50 g of Apiguard (Vita Bee Health, Basingstoke, UK) prior to cold storage.

### *Nosema* Spores per Bee

*Nosema* samples were processed by placing the bees in a 50 ml test tube containing 20 ml of ultra-pure water. The bees were ground and homogenized with a Brinkmann Polytron Kinematica Generator PT-DA 2120/2 WEC (Kinematica AG 6102 Malters Switzerland) for 5 s. The homogenizer was double rinsed using a 500 ml flask with distilled water. Samples were allowed to equilibrate for 30–60 s. The supernatant was removed from the center of the sample in the test tube (clear area) and placed into a 1.5 ml Eppendorf tube, and frozen until counted for *Nosema* spores.

Samples of the supernatant were thawed prior to analysis and vortexed. A 15 µl sample was transferred to a Bright-Line hemocytometer (Hausser Scientific, Horsham, PA, USA), and spores were counted using a compound microscope (Leica CME, Buffalo, NY, USA) at 400× with phase-contrast lighting. *Nosema* spores were counted in 16 small squares within 5 larger squares. The final spore count was calculated by multiplying the hemocytometer counts by 50,000 (Fries et al. 2013).

### Fat Body Analysis

Fat body metrics were measured using protocols described in DeGrandi-Hoffman et al. (2023). Briefly, 10 worker bees from each colony (year 1: *n* = 39, year 2: *n* = 88) were placed onto a block of dry ice, and their abdomens were removed and placed into a waxed petri dish ventral side up. The entire gut was removed, and the remaining abdominal carcass with the fat body attached was rinsed to remove the remaining gut contents, blotted dry, and placed in a 2ml tube. The 10 abdomens and 3 chrome beads (3.2 mm) were placed in pre-weighed reinforced polypropylene 2mL tubes (XXTuff Microvials, Bio Spec Products catalog number 330TX, Bio Spec Products, Bartlesville, OK, USA). Samples were placed into a −20 °C freezer until further processing.

Fat body analysis began by placing samples into a Fisher Scientific Iso Temp oven (Thermo Fisher Scientific, Waltham, MA, USA) set at 60 °C until dry (4 days). Samples were removed, and a new previously weighed lid for the tube was immediately applied. The preliminary weight of the tube with beads was subtracted from the dry weight to determine the dry weight of 10 abdominal carcasses. Samples were placed into a −20 °C freezer until processed for protein and lipid concentrations.

Prior to protein and lipid analyses, 1 ml of PBS was added to each sample vial. The sample was macerated with a Bio Spec Mini-Beadbeater 96 (Bio Spec Products, Distributor Cole Parmer, Vernon Hills, IL, USA) for 60 s. Samples were then centrifuged (Eppendorf Centrifuge 5424, Hamburg, Germany) at 15,000 rpm for 6 min. Without disturbing the pellet, 100 µl of the supernatant was removed from the tube, just below the top layer of lipids, and was placed into a tube with 900 µl of a PBS, Halt mixture (49.6 ml of PBS and 400 µl of Halt) and 1% EDTA-free Halt Protease Inhibitor Cocktail (#78437, Thermo Scientific, Rockford, IL, USA). The sample was frozen until processed for protein. The remaining sample was frozen until lipid analysis.

### Protein Analysis

Protein concentrations in the fat body were analyzed using a BCA Protein Assay kit (Thermo Scientific product 23225, Waltham, MA, USA) according to methods described in DeGrandi-Hoffman et al. (2023) and the manufacturer instructions. Samples were run in triplicate and read in a Bio Tek Synergy HT Microplate reader (BioTek Instruments, Winooski, VT, USA) at a wavelength of 562 nm. The blank wells absorbance value was subtracted from each standard and sample. A second-order polynomial curve of the standards was used to determine protein concentration in µg/ml. Because the fat body sample had a high concentration of protein, it was plated at a 3% solution. Protein in fat body samples was estimated first by evaluating the absorbance against that of a standard curve and then correcting for the sample dilution made prior to analysis.

### Lipid Analysis

Lipid analysis was conducted using methods described in DeGrandi-Hoffman et al. (2023). Briefly, samples were removed from the −80 °C freezer and thawed before processing. Sample tubes were vortexed and poured into Fisher Brand 10 ml disposable 13 mm × 100 mm culture tubes (Thermo Fisher Scientific, Waltham, MA, USA), and the original sample tube was washed with an additional 1 ml dH_2_O also added to the original sample tube. The sample was vortexed and poured into the culture tube. A 2:1 mixture of chloroform:methanol (1 ml) was added to the culture tube along with 210 µl of 0.25% KCI. The sample was vortexed and then centrifuged at 2,000 rpm for 15 min (Thermo Scientific Sorvall ST 16 centrifuge, Thermo Fisher, Waltham, MA, USA). The bottom chloroform layer was removed and placed into a new 2 ml glass screw cap vial (Sun Sri Part # 500 306, Sun SRI, Rockwood, TN, USA), and the process was repeated. Standards consisted of serial dilutions of corn oil (750 µg/µl) dissolved in chloroform. The negative control consisted of 100 µl of chloroform. Samples, standards, and the negative control were dried to completion for approximately 1.5 h (Savant SPD 2010 Speedvac Concentrator, Thermo Fisher Scientific, Waltham, MA, USA). Dried samples were reacted with 182 µl of concentrated sulfuric acid at 100 °C for 15 min (Heating block Model HP30A, Torrey Pines Scientific, Carlsbad, CA, USA) and then 1,478 µl of vanillin-phosphoric acid for 15 min at room temperature in the dark. Samples were diluted, so they were within the range of the standard curve. Each of the negative controls, standards, and samples (100 µl) were plated in triplicate and read using a spectrophotometer at 525 nm (Agilent Bio Tek Synergy HT, Santa Clara, CA, USA). The average absorbance value for the negative control was subtracted from each standard and sample. A linear curve of the standards was used to infer the µg/µl of lipid per sample.

### Statistical Analysis

All statistical analyses were conducted using Minitab (Minitab, LLC, State College, PA, USA) and JMP (SAS Institute, Cary, NC, USA) statistical software packages unless otherwise noted. In year 1, data were organized into 2 groups based on *Nosema* spore number: colonies averaging less than (<) 1 million (1 × 10^6^) spores per bee and colonies averaging greater than (>) 1 × 10^6^ spores per bee prior to cold storage. Combs with bees before and after cold storage and after almond bloom were compared between the 2 spore groups using repeated measures analysis of variance. Percentages of colonies that survived cold storage (≥4 combs covered with bees), could be rented for almond pollination (≥6 combs covered with bees), and were alive after almond bloom (≥4 combs covered with bees) were compared between the 2 groups using Fisher’s Exact test (Hoffman 2015). Combs of brood after cold storage and almond bloom were compared between the spore groups using t-tests. Relationships between colony size and spore numbers before and after cold storage and almond bloom were conducted using linear regression. *Nosema* spore counts were log_10_ (*n* + 1) transformed prior to analysis. Only colonies where *Nosema* was detected were included in the analysis. Fat body weight, lipid, and protein concentration before and after cold storage were compared between colonies averaging either < or >1 × 10^6^ spores per bee using analysis of variance with sample time and spore group as factors in the general linear model.

In year 1, comparisons were made of mites per 100 bees prior to cold storage between colonies in the spore groups using a *t*-test. In year 2, comparisons of mites per 100 bees were made among the 4 treatment groups with a one-way analysis of variance. The mite data were not normally distributed and were transformed (1 + square root (*n*)) prior to analysis. In year 2, the proportion of colonies testing positive for *Nosema* before and after treatments were compared within each treatment group using Fisher’s exact test.

In year 2, average spores per bee in pre- and post-treatment samples were compared within each treatment group using t-tests. The proportion of colonies with >1 × 10^6^ spores per bee during the pretreatment sample was compared among treatments using Marascuilo’s Procedure (Engineering Statistics Handbook https://www.itl.nist.gov/div898/handbook/index.htm). After Fumagillin or control treatment, average spores per bee were compared among the groups using one-way analysis of variance. Data were transformed (1 + square root (*n*)) for normality. We tested for a relationship between initial and pre-cold storage *Nosema* spore numbers in control (C-SUP and C-POL) groups using linear regression. Only colonies where *Nosema* was detected were used in the analyses. Comparisons of slope and y-intercepts were made between C-POL and C-SUP regression equations. Multifactor linear regression was used to test for relationships between pre-cold storage factors (colony sizes and *Nosema* spore numbers) and post-cold storage and almond bloom colony sizes. Only colonies where spores were detected were used in the analyses. Spore numbers were log_10_ transformed prior to analysis. Binary logistic regression with control colony data (C-POL and C-SUP) was used to test for relationships between surviving cold storage (≥4 frames with bees), being large enough for almond pollination rental (>6 frames of bees for colony rental) and having ≥4 frames with bees after bloom based on pre-cold storage *Nosema* spores per bee and colony size (Fritz and Berger 2015).

We grouped C-POL and C-SUP colonies based on pre-cold storage spore numbers into those averaging zero, >0 <1 × 10^6^, or ≥1 × 10^6^ spores per bee prior to cold storage. We chose the groupings so we could make comparisons among colonies with no *Nosema* and those with moderate and severe infections. We compared colony sizes among the groups before and after cold storage and almond bloom using two-way analysis of variance with time and spore group as factors in the general linear model. Separate analyses were conducted for C-POL and C-SUP colonies, and then data were combined to test for time, diet, and spore group effects. Percentages of colonies that survived cold storage could be rented for almond pollination and were alive after almond bloom were compared among the 3 groups using Marascuilo’s Procedure.

We explored possible effects of Fumagillin by comparing the sizes of control colonies with zero spores prior to cold storage (i.e., post-treatment samples) with those fed the same diet and having zero spores after Fumagillin treatment (C-POL vs. F-POL and C-SUP vs. F-SUP). We compared combs with bees and brood after cold storage and almond bloom using analysis of variance with treatment and sample times as factors in the general linear model.

Fat body metrics (dry weight, lipid, and protein concentrations) were compared among treatment groups before and after cold storage using analysis of variance with time and diet as factors in the general linear model. Comparisons of fat body metrics based on average *Nosema* spores per bee were made in a similar manner to colony size. Pre- and post-cold storage C-POL and C-SUP fat body measurements from groups averaging zero, >0 <1 × 10^6^, or ≥1 × 10^6^ spores per bee were compared using two-way analysis of variance with time and group as factors in the general linear model. A final analysis was conducted with combined data from both diets to test for the effects of diet in addition to spore number on fat body metrics. In addition, fat body weight, protein and lipid concentrations were compared using t-tests between colonies averaging zero spores per bee and either treated with Fumagillin or sugar syrup alone. Separate analyses were conducted for each diet.

## Results

### Varroa Mites per 100 Bees

In year 1, colonies averaged <1 Varroa mite per 100 bees in September (0.17 ± 0.08, *n* = 39). Average mites per 100 bees did not differ between colonies averaging < 1 × 10^6^ or > 1 × 10^6^ spores per bees (< 1 × 10^6^ spores = 0.88 ± 0.47, > 1 × 10^6^ spores = 0.64 ± 0.24, *t* = 0.47, *df* = 24, *P* = 0.645). In year 2, mites per 100 bees ranged from 0.03 ± 0.02 (F-POL and F-SUP) to 0.07 ± 0.03 (C-POL and C-SUP) on September 1. Mite numbers did not differ among the treatment groups (*F* = 0.66, *df* = 3,348, *P* = 0.58).

### *Nosema* Spores per Bee

**Fig. 2.**
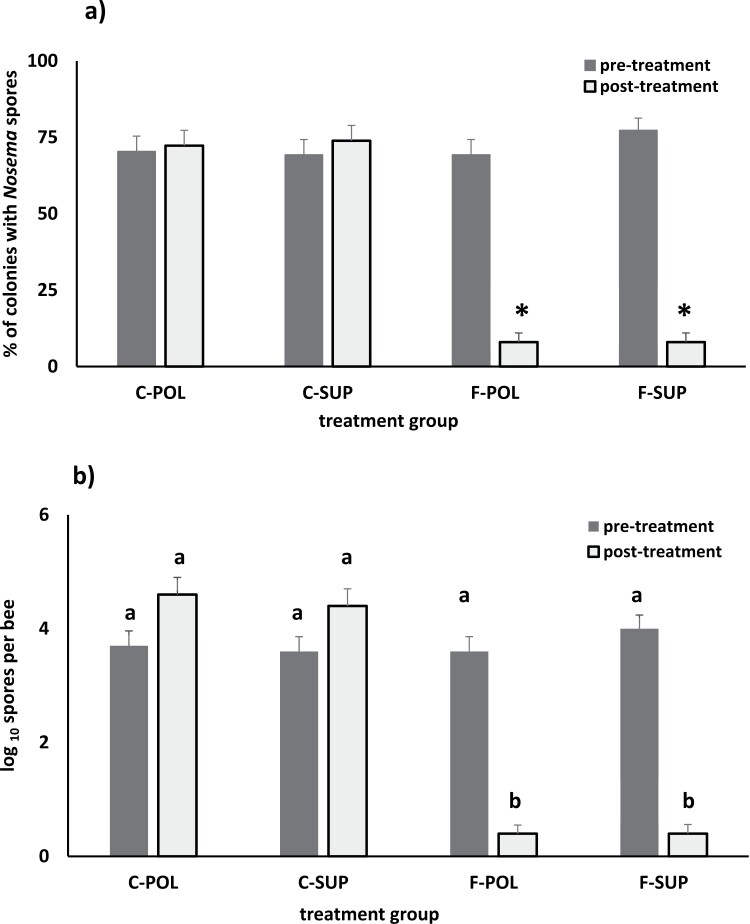
Percentages of colonies where *Nosema spp.* spores were detected (a) and the average number of spores per bee (b) in 4 treatment groups (n = 88 colonies per group) assigned by diet and application of Fumidil-B (Fumagillin). F-POL and F-SUP were treated with Fumagillin in sugar syrup and fed either pollen (F-POL) or protein supplement (F-SUP). C-POL and C-SUP were fed sugar syrup alone and provided with either pollen (C-POL) or protein supplement (C-SUP). The percentage of control colonies with no detectable spores in the pre- and post-treatment samples did not differ between C-POL and C-SUP (C-POL = 27.3% and C-SUP = 26.1%; *z* = 0.17, *P* = 0.865). Significantly fewer F-POL and F-SUP had detectable spores after treatment compared with pretreatment (*z* = 0.58, *P* = 0.56). The percentage of colonies with *Nosema* spores that differ significantly between the pre- and post-treatment sampling intervals in each treatment group is indicated with an asterisk (*). Average numbers of spores per bee were log_10_ transformed prior to analysis and did not differ among the groups prior to treatment (*F*_3,348_ = 0.67, *P* = 0.57) B). Spore numbers in C-POL and C-SUP did not differ between pre- and post-treatment intervals (C-POL: *t*_171_ = 1.63, *P* = 0.10; C-SUP: *t*_173_ = 1.59, *P* = 0.11). In F-POL and F-SUP, post-treatment spore numbers were significantly lower than pretreatment (F-POL: *t*_140_ = 10.8, *P* < 0.0001; F-SUP: *t*_151_ = 12.7, *P* < 0.0001). Post-treatment averages of log_10_ spores per bee with the same letter do not differ as determined by analysis of variance followed by a Tukey’s multiple comparison test (*F*_3,348_ = 95.3, *P* < 0.0001).

In year 1, average pre-cold storage spore numbers in colonies ranged from 0 to 28.5 × 10^6^ spores per bee. *Nosema* spores were detected in 67% of colonies prior to cold storage. Of the colonies with spores, 38% had < 1 × 10^6^ spores and 62% had > 1 × 10^6^ spores. In year 2, *Nosema* spores were detected in 70.4% of C-POL, 69.3% of C-SUP, and F-POL and 77.3% of F-SUP in the pretreatment samples (Fig. 2). Average spores per bee during pretreatment sampling did not differ among the treatments. However, the proportion of colonies that had ≥ 1 × 10^6^ spores per bee differed among the groups (*χ*^2^ = 6.78, *P* = 0.009; C-POL = 0.01, C-SUP = 0.10, F-POL = 0.03, F-SUP = 0.04). After Fumagillin treatment, *Nosema* spores were detected in significantly fewer colonies than in the untreated controls (8% of F-POL and F-SUP vs. 72%–73% of C-POL and C-SUP). Percentages of C-POL and C-SUP with *Nosema* did not change between the pretreatment (1 Sep) and post-treatment samples (27 Sep) nor did the percentage of control colonies that had no detectable spores (C-POL: 27.3%, C-SUP: 26.1%). C-POL, however, had a significantly higher percentage of colonies averaging more than 1 × 10^6^ spores per bee (C-POL = 54.5%, C-SUP = 39.8%; *z* = 1.98, *P* = 0.047) in the post-treatment sample. For colonies treated with Fumagillin, 92% of both F-POL and F-SUP had no detectable spores when hives were put into cold storage; 1.1% of F-POL and 2.3% of F-SUP averaged more than 1 × 10^6^ spores per bee (*z* = 0.58, *P* = 0.56).

In year 2, average spores per bee during pretreatment sampling ranged from 0 to 4.65 × 10^6^ and did not differ among treatment groups. After treatment, spore counts were lower in Fumagillin treated colonies compared with untreated controls. C-POL and C-SUP spore counts increased, though not significantly, between pre- and post-treatment counts and ranged from 0 to 29.6 × 10^6^ spores per bee. There was a significant relationship between initial and pre-cold storage spore numbers for both C-POL and C-SUP (Table 1). Comparison of C-POL and C-SUP regression lines indicated no difference in slope (*P* = 0.887) or y-intercept (*P* = 0.728), indicating that the rate of increase in spore numbers was not affected by diet.

**Table 1.** Relationship between initial counts of *Nosema* spores per bee (September 1) and counts (3 wks later) prior to colonies entering cold storage for overwintering during year 2 of the study as determined by linear regression. Colonies were not treated for *Nosema* and were fed either pollen (C-POL) or protein supplement (C-SUP) prior to cold storage. All spore numbers were log_10_ transformed prior to analysis

Treatment group	Y-intercept (standard error)	Slope (standard error)	*df*	*P* y-intercept	*P* slope	*R* ^2^
C-POL	1.72 0.43)	0.773 (0.10)	1, 1, 86	<0.0001	<0.0001	41.6
C-SUP	1.68 (0.39)	0.754 (0.09)	1, 1, 86	<0.0001	<0.0001	45

### Colony Size and Survival

**Fig. 3.**
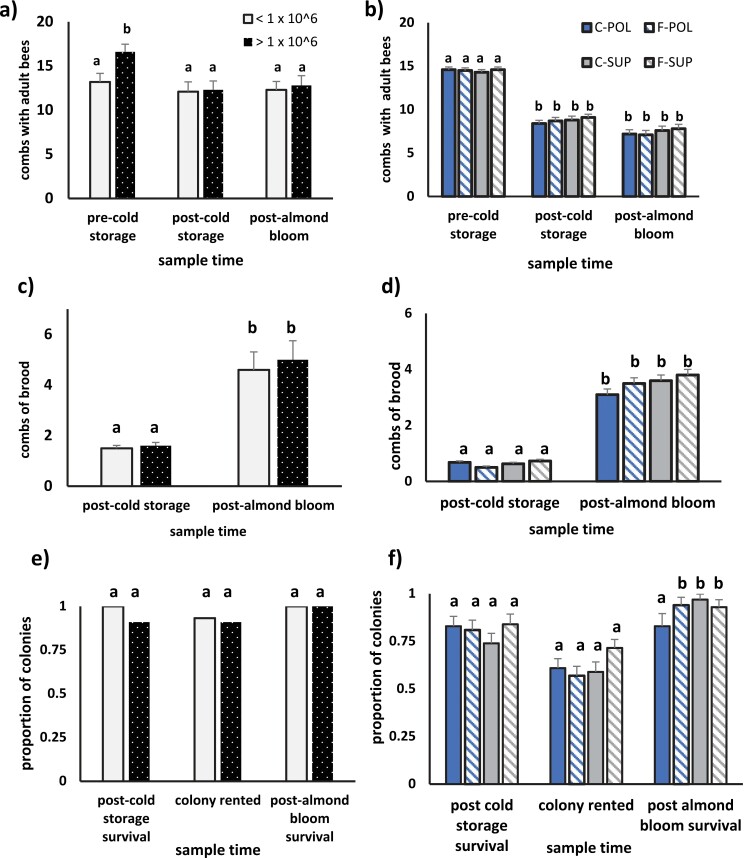
Average combs with adult bees in years 1 (a) and 2 (b), combs of brood in years 1 (c) and 2 (d) and the proportion of colonies in years 1 (e) and 2 (f) that survived overwintering in cold storage were large enough to be rented for almond pollination and were alive after bloom. In year 1, colonies were grouped according to the average number of *Nosema* spores per bee (< or > 1 × 10^6^; *n* = 16 and 23, respectively). Combs with bees were affected by time (*F*_1, 111_ = 3.81, *P* = 0.025) but not average *Nosema* spores per bee (*F*_2, 111_ = 2.37, *P* = 0.13). Interaction terms were not significant (*F*_2, 111_ = 1.36, *P* = 0.26). Combs of brood were affected by time (*F*_1, 74_ = 90.1, *P* < 0.0001) but not average spores per bee (*F*_1, 74_ = 1.51, *P* = 0.22). Interaction terms were not significant (*F*_1, 74_ = 0.68, *P* = 0.41). Post-cold storage survival did not differ between colonies with < or > 1 × 10^6^ spores per bee (*P* = 0.51). Similar proportions of colonies could be rented for almond pollination (p = 1.0) and were alive after almond bloom (*P* = 1.0). In year 2, colonies were either treated for *Nosema* spp. with Fumagillin (Fumidil-B) mixed in sugar syrup or fed sugar syrup without Fumagillin (controls) prior to overwintering in cold storage. Treated colonies were fed either pollen (F-POL) or protein supplement (F-SUP) as were untreated control colonies (pollen—C-POL or protein supplement—C-SUP) (*n* = 88 per treatment group). Averages for combs with bees among treatment groups were affected by sample time (time: *F*_2, 348_ = 512.1, *P* < 0.0001) but not *Nosema* treatment (*F*_1, 349_ = 0.056, *P* = 0.81) or diet (*F*_1, 349_ = 1.16, *P* < 0.28). Combs of brood after cold storage and almond bloom did not differ between diets (*F*_1, 345_ = 0.04, *P* = 0.843) or *Nosema* treatment (*F*_1, 345_ = 0.027, *P* < 0.60) but did differ between sample times (*F*_1, 345_ = 770.5, *P* < 0.0001). The proportion of colonies that survived cold storage and were rented for almond pollination was similar among treatments (post-cold storage survival: *χ*^2^ = 3.51, *df* = 3, *P* = 0.32; colony rented: *χ*^2^ = 4.77, *df* = 3, *P* = 0.19). Post-bloom survival differed among the groups (*χ*^2^ = 12.6, *P* = 0.005), with significantly lower survival for C-POL than the other groups (*χ*^2^ = 8.81, *P* = 0.032), which did not differ. Averages and proportions within the same year labeled with the same letter are not significantly different at the *P* = 0.05 level as determined by either Tukey’s multiple comparison test (averages) or Marascuilo’s Procedure (proportions).

In year-1, post-cold storage and almond bloom colony sizes were significantly related to the time when combs were measured but not *Nosema* spore numbers (Supplementary Table S1). In pre-cold storage measurements, colonies averaged 13.3–16.6 combs with bees (< or > 1 × 10^6^ spores per bee respectively) (Fig. 3). After cold storage and almond bloom, colonies were smaller (12.1–12.8 combs with bees) but did not differ between treatment groups in size or survival.

All colonies in year-1 produced brood while in cold storage and throughout almond bloom. The amount of brood was not affected by *Nosema* spore number. Colonies had significantly more brood after almond bloom than after cold storage. Similar percentages of colonies averaging < or > 1 × 10^6^ spores per bee survived cold storage and were large enough to rent for almond pollination. All rented colonies were alive after almond pollination.

In year 2, colony survival after cold storage was between 74% and 84% and did not differ among the 4 treatment groups (Fig. 3). Percentages of colonies that could be rented for almond pollination also were similar among the 4 treatments, but fewer C-POL colonies were alive after almond bloom compared with the other treatments. Colonies had an average of 14.3–14.6 combs with bees when they were put into cold storage and 8.4–9.1 combs with bees when they were removed from the facilities. Averages for combs with bees were affected by sample time but not diet or *Nosema* treatment. After almond bloom, colonies ranged from 7.2 to 7.8 combs with bees. Combs of brood after cold storage and almond bloom were significantly affected by sample time but not *Nosema* treatment or diet.

Post-cold storage and almond bloom C-POL and C-SUP colony sizes were significantly related to pre-cold storage combs with adult bees but not *Nosema* spore numbers (Supplementary Table S1). The proportion of colonies that survived cold storage (≥4 frames with bees) and were alive after almond bloom also was significantly related to pre-cold storage colony size but not average *Nosema* spores per bee for C-POL or C-SUP (Table 2). The proportion of C-POL and C-SUP that were large enough to rent for almond pollination (>6 frames with bees after cold storage) however, was significantly related to both pre-cold storage colony size and spores per bee.

**Table 2.** Binary logistic regression of the relationship between colony survival after cold storage (≥4 frames of bees), suitability for almond pollination rental (≥6 frames with bees), and survival after almond bloom (≥4 frames of bees) as a function of *Nosema* spores per bee and colony size (combs with bees) prior to cold storage. Colonies were fed either pollen patty or protein supplement prior to cold storage

Diet treatment	Response	Predictor(s)	Coefficient	Standard error	*df*	*P*	Goodness of fit (Pearson)		
							*df*	*χ* ^2^	*P*
Pollen patty	Colony survival	Spores per bee	−0.136	0.107	1	0.201			
		Colony size	0.162	0.044	1	<0.0001			
		Error			86		86	84.22	0.534
	Colony rental	Spores per bee	−0.182	0.083	1	0.028			
		Colony size	0.097	0.032	1	0.002			
		Error			86		86	87.55	0.433
	Post-bloom survival	Spores per bee	−0.161	0.082	1	0.049			
		Colony size	0.094	0.032	1	0.003			
		Error			86		86	87.42	0.437
Protein supplement	Colony survival	Spores per bee	−0.119	0.104	1	0.252			
		Colony size	0.153	0.042	1	<0.0001			
		Error			86		86	87.05	0.448
	Colony rental	Spores per bee	−0.214	0.085	1	0.012			
		Colony size	0.101	0.032	1	0.002			
		Error			86		86	88.81	0.396
	Post-bloom survival	Spores per bee	−0.045	0.092	1	0.625			
		Colony size	0.114	0.035	1	0.001			
		Error			86		86	84.91	0.513

### *Nosema* Infection Levels and Colony Size and Survival

**Fig. 4.**
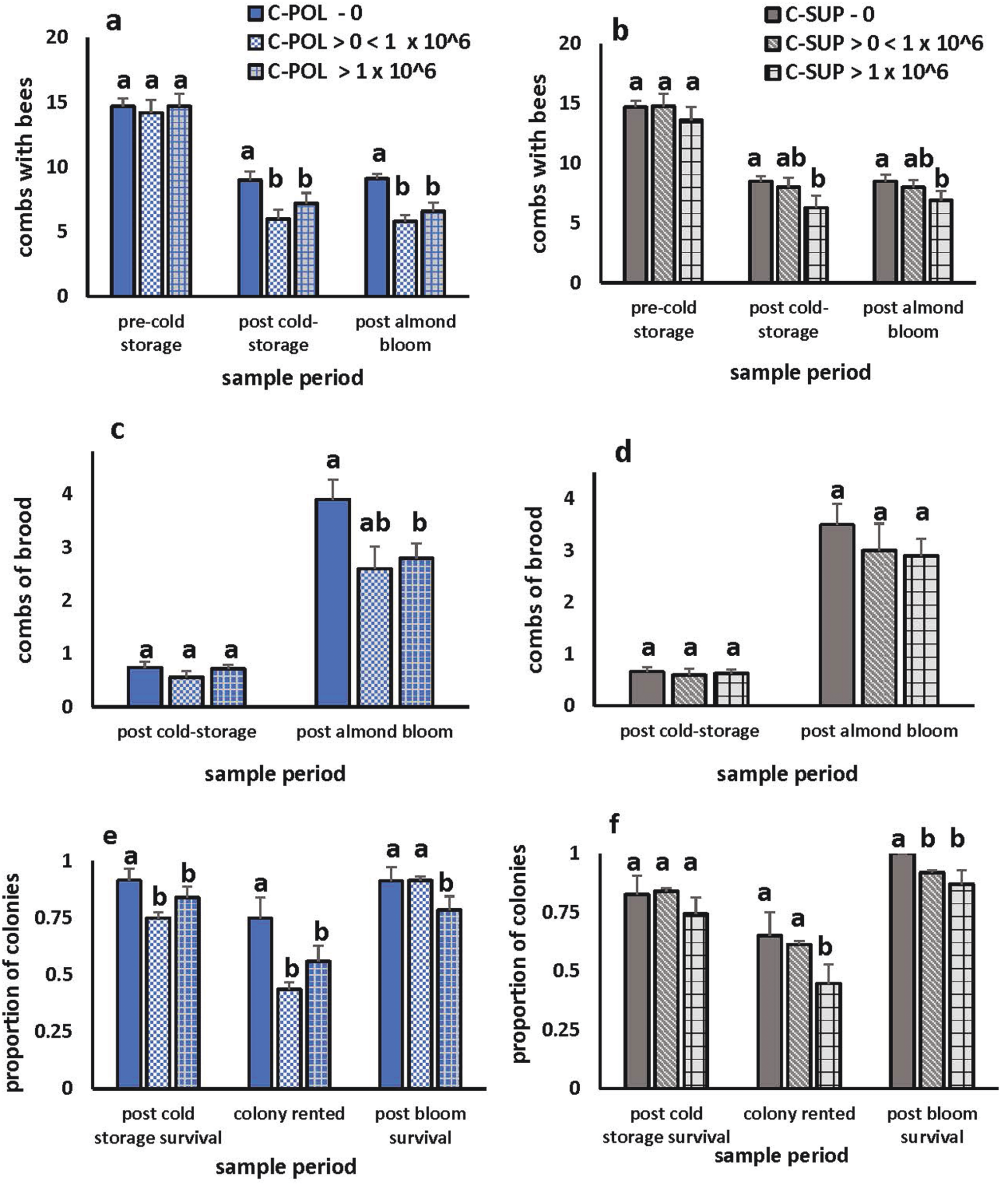
Combs with bees (a) and (b) and brood (c) and (d) in colonies fed either pollen patty (C-POL) or protein supplement (C-SUP) prior to overwintering in cold storage. Colonies averaged zero (0), > 0 < I x 10^6^, or > 1 × 10^6^*Nosema* spores per bee prior to cold storage. Averages of combs with bees in C-POL colonies were significantly affected by pre-cold storage spore number (*F*_2,170_ = 7.02, *P* = 0.001) but not sample period (*F*_1,170_ = 0.09, *P* = 0.76) or their interaction (*F*_2,170_ = 0.11, *P* = 0.895). Similar results occurred in C-SUP where average combs of bees were significantly affected by spore number (*F*_2,170_ = 3.53, *P* = 0.031) but not sample period (*F*_1,170_ = 0.001, *P* = 0.968) or their interaction (*F*_2,170_ = 0.09, *P* = 0.915). An analysis combining data from both diet treatments indicated significant effects of spore numbers (*F*_2,347_ = 7.44, *P* = 0.001) but not sample period (*F*_1,347_ = 0.05, *P* = 0.826) or diet treatment (*F*_1,347_ = 0.23, *P* = 0.630) on combs with bees. Average frames of brood in C-POL were affected by sample period (*F*_1,123_ = 120.1, *P* < 0.0001) and spore number (*F*_2,123_ = 4.39, *P* < 0.014). Frames of brood in C-SUP were affected by sample period (*F*_1,123_ = 111.83, *P* < 0.0001) but not spore number (*F*_2,123_ = 0.88, *P* < 0.417). An analysis combining data from both diet treatments indicated significant effects of spore numbers (*F*_2,309_ = 4.07, *P* = 0.018) and sample period (*F*_1,309_ = 242.8, *P* =  < 0.0001) but not diet (F_1,309_ = 0.02, p = 0.882) on brood production. Means labeled with the same letter for each sample period are not significantly different at *P* = 0.05 as determined by Tukey’s multiple comparison test. Proportions of C-POL colonies surviving cold storage (*χ*^2^ = 1.66, *df* = 2, *P* = 0.43), rented for almond pollination (*χ*^*2*^ = 18.6, *df* = 2, *P* < 0.0001), and alive after almond bloom (*χ*^2^ = 8.98, *df* = 2, *P* = 0.011) (e). Proportions of C-SUP colonies alive after cold storage (*χ*^2^ = 3.50, *df* = 2, *P* = 0.17), rented for almond pollination (*χ*^2^ = 6.54, *df* = 2, *P* = 04), and alive after almond bloom (*χ*^2^ = 12.4, *df* = 2, *P* = 0.002) (f). Proportions labeled with the same letter are not significantly different as determined by Marascuili’s Procedure for comparing multiple proportions.

In year 2, we determined if colony size and survival differed with levels of *Nosema* infection and diet treatment by separating control colonies into groups with zero, > 0 < 1 × 10^6^, or ≥ 1 × 10^6^ spores per bee prior to cold storage. C-POL and C-SUP had similar percentages of colonies where no *Nosema* spores were detected prior to cold storage (C-POL: 27.3% and C-SUP 26.1%). C-POL had 17 colonies (19.3%) averaging > 0 and < 1 × 10^6^ and 48 (53.4%) averaging ≥ 1 × 10^6^. C-SUP had 30 colonies (34.1%) with > 0 < 1 × 10^6^ and 35 (39.8%) with ≥ 1 × 10^6^ prior to cold storage. Though C-POL colonies in each spore group were similar in size prior to cold storage, colonies with zero spores were significantly larger after cold storage and almond bloom than those where spores were detected (Fig. 4). Results were similar for C-SUP.

Combs of brood after cold storage in C-POL did not differ among the spore groups, but after almond bloom, colonies with zero spores had more 1.1 more combs of brood than those averaging > 1 × 10^6^ spores per bee. Brood areas in C-SUP colonies were similar among the spore groups after cold storage and almond bloom. Combined C-POL and C-SUP data indicated no significant effects of diet on brood areas.

The proportion of C-POL colonies that were large enough to rent for almond pollination and were alive after almond bloom was highest for those with zero spores. Similarly, more C-SUP colonies with zero spores could be rented for pollination and were alive after almond bloom than those with > 1 × 10^6^ spores.

### Effect of Fumigillan Treatment on Colony Size and Survival After Cold Storage and Almond Bloom

There was a significant effect of Fumagillin treatment on the size of colonies fed pollen (Table 3). Though combs of bees were similar prior to cold storage, F-POL were smaller than C-POL after cold storage and almond bloom. Fumagillin treatment did not significantly affect F-SUP colony sizes, which were only affected by sample time. Analysis of Fumagillin treatment on brood production indicated significant effects of sample time and treatment with less brood produced in F-POL compared with C-POL colonies. Fumagillin treatment did not affect brood production in colonies fed protein supplements as only sample time was significant.

**Table 3.** Combs with bees and combs of brood in colonies with zero *Nosema spores* prior to cold storage. Colonies were fed either pollen (POL) or protein supplement (SUP) and treated with Fumidil-B (Fumagillin) in sugar syrup (F-POL and F-SUP) prior to cold storage or with sugar syrup alone (C-POL and C-SUP). Frames with bees were measured 3 wk after treatment (pre-cold storage), after colonies were removed from cold storage (post-cold storage), and after almond bloom (post-bloom). Colonies did not contain brood during the pre-cold storage sample period. Data for frames with bees were analyzed using repeated measures analysis with sample time, and ±Fumagillin treatment in sugar syrup prior to cold storage as factors in the model. Frames of brood were compared between the sample times and treatments using a general linear model

Response	Sample time			Factor	*F*	*df*	*P*
	Pre-cold storage	Post-cold storage	Post-bloom				
**Combs with bees**							
C-POL	14.7 ± 0.5	8.8 ± 0.8	9.1 ± 0.9	Sample time	65.6	2	<0.0001
F-POL	14.4 ± 0.3	7.0 ± 0.4	7.2 ± 0.5	±Treatment	7.0	1	0.009
				Sample time * ±treatment	1.04	2	0.36
				Error		311	
C-SUP	14.7 ± 0.6	8.8 ± 1.0	8.5 ± 1.1	Sample time	60.0	2	<0.0001
F-SUP	14.6 ± 0.3	8.0 ± 0.4	7.8 ± 0.5	±Treatment	1.03	1	0.31
				Sample time * ±treatment	0.18	2	0.84
				Error		308	
**Combs with brood**							
C-POL	na	0.71 ± 0.1	3.8 ± 0.4	Sample time	140.3	1	<0.0001
F-POL	na	0.49 ± 0.05	3.1 ± 0.2	±Treatment	3.74	1	0.05
				Sample time * ±treatment	1.03	1	0.31
				Error		205	
C-SUP	na	0.63 ± 0.05	3.6 ± 0.22	Sample time	129.9	1	<0.0001
F-SUP	na	0.73 ± 0.06	3.4 ± 0.2	±Treatment	0.08	1	0.77
				Sample time * ±treatment	0.01	1	0.91
				Error		203	

### Effect of *Nosema* and Fumagillin Treatment on Fat Body

In year 1, fat body weights, lipid, and protein concentrations before and after cold storage did not differ between colonies, averaging < 1 × 10^6^ or > 1 × 10^6^ spores per bee (Supplementary Table S2).

Pre-cold storage fat body metrics differed significantly from post-cold storage. Fat body weight and protein concentration increased significantly while bees were in cold storage, and lipid concentrations significantly decreased.

**Fig. 5.**
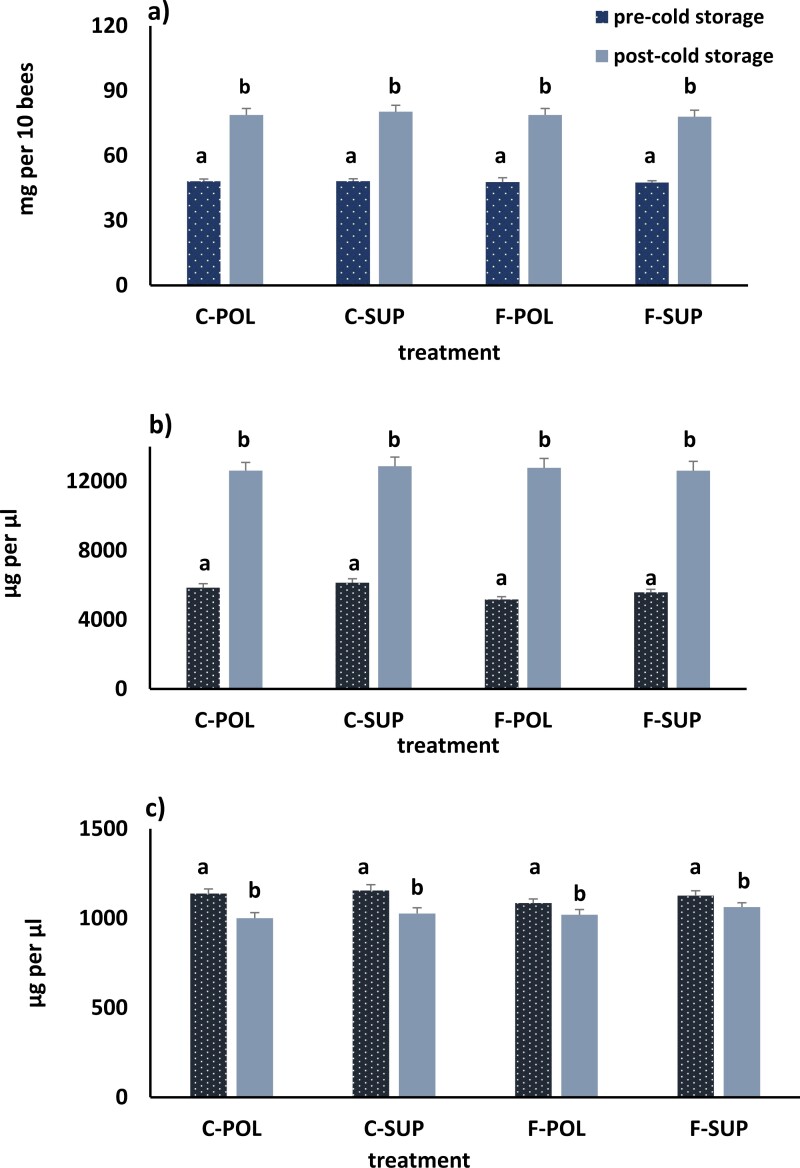
Average fat body weights (a), protein (b) and lipid (c) concentrations in honey bees before and after overwintering in cold storage. Colonies (*n* = 88 per treatment group) were either treated for *Nosema* spp. with Fumagillin (Fumidil-B) mixed in sugar syrup or fed sugar syrup without Fumagillin (controls) 3 wk prior to overwintering. Colonies treated with Fumagillin were fed either pollen (F-POL) or protein supplement (F-SUP) as were untreated control colonies (pollen—C-POL or protein supplement—C-SUP). Fat body weights, protein, and lipid concentrations differed between sampling times but not between diets or whether colonies had Fumigillan treatments (±FUM) (weight: *F*_(sample time)_ = 329.5, *P* < 0.0001; *F*_(diet)_ = 0.01, *P* = 0.931; *F*_(±FUM)_ = 0.24, *P* = 0.623, *df* = 1, 1, 1, 693; protein concentrations: *F*_(sample time)_ = 632.75, *P* < 0.0001, (*F*_(diet)_ = 0.51, *P* = 0.48; *F*_(±FUM)_ = 1.45, *P* = 0.23, *df* = 1, 1, 1, 693; lipid concentration: *F*_(sample time)_ = 22.88, *P* < 0.0001; *F*_(diet)_ = 2.2, *P* = 0.138; *F*_(±FUM)_ = 0.01, *P* = 0.919, *df* = 1, 1, 1, 693). Means with the same letter are not significantly different *P* = 0.05.

Similar to year 1, fat body weight, protein, and lipid concentration in year 2 changed significantly while bees were in cold storage. Weights increased as did protein concentration while lipid concentrations decreased (Fig. 5). Analysis of individual sample times showed that pre-cold storage protein concentrations differed among treatments, with F-POL levels being lower than C-POL and C-SUP. Fat body weight and lipid concentrations did not differ among treatments. Post-cold storage fat body weight, lipid, and protein concentrations did not differ among treatment groups.

Analysis of pre- and post-cold storage fat body metrics based on grouping by spore number (zero, > 0 < 1 × 10^6^ and > 1 × 10^6^ spores per bee) indicated that fat body weight, protein, and lipid concentrations for C-POL were affected only by sample time and not spore number (Table 4). Interaction terms also were not significant. Similar results were found with C-SUP, except that interaction terms (sample time × spore number) for fat body weight and protein concentrations were significant. A final analysis that combined data from both diet treatments indicated that diet effects were not significant. Only sample time significantly affected fat body metrics.

**Table 4.** Comparisons of fat body dry weights, protein, and lipid concentrations in worker honey bees from colonies averaging zero, >0 <1 × 10^6^ or ≥1 × 10^6^*Nosema* spores per bee prior to overwintering in cold storage. Colonies were fed either pollen (C-POL) or protein supplement (C-SUP) prior to overwintering. Fat body measurements were made using samples taken before (pre-cold storage) and after (post-cold storage) overwintering. Averages are ± standard error. Average weights are the sum of 10 honey bee fat bodies per colony. Sample sizes (*n*) represent the number of colonies and are shown in parentheses beneath the average

Treatment	Fat body metric	Sample time	Average ± SE (per spore group)			Source	*F*	*df*	*P*
			0 (*n*)	>0 < 1 × 10^6^ (*n*)	≥10^6^ (*n*)				
C-POL	Weight (mg)	Pre-cold storage	48.6 ± 1.8 (23)	47.1 ± 2.0 (17)	48.0 ± 1.7 (47)	Sample time	70.7	1	<0.0001
		Post-cold storage	79.3 ± 5.4 (23)	79.1 ± 6.5 (17)	78.4 ± 4.7 (47)	Spore group	0.02	2	0.98
						Interaction	0.02	1	0.98
						Error		173	
	Protein concentration (µg/µl)	Pre-cold storage	6363.0 ± 505 (23)	6466.0 ± 557.0 (17)	5374.0 ± 325.0 (47)	Sample time	126.7	1	<0.0001
		Post-cold storage	12411.0 ± 913.0 (23)	12745 ± 1142.0 (17)	12769.0 ± 634.0 (47)	Spore group	0.33	2	0.72
						Interaction	0.69	2	0.50
						Error		173	
	Lipid concentration	Pre-cold storage	1149.3 ± 67.9 (23)	1168.7 ± 50.9 (17)	1121.4 ± 35.4	Sample time	10.1	1	0.002
		Post-cold storage	1027.3 ± 60.6 (23)	993.5 ± 68.4 (17)	978.0 ± 43.7 (47)	Spore group	0.36	2	0.70
						Interaction	0.09	1	0.91
						Error		173	
C-SUP	Weight (mg)	Pre-cold storage	48.2 ± 2.4 (23)	47.6 ± 2.2 (29)	48.6 ± 1.3 (35)	Sample time	82.6	1	<0.0001
		Post-cold storage	67.7 ± 6.3 (23)	89.5 ± 4.4 (28)	80.6 ± 5.5 (35)	Spore group	2.48	2	0.151
						Interaction	3.89	1	0.002
						Error		169	
	Protein concentration (µg /µl)	Pre-cold storage	6757.0 ± 516.0 (23)	5899.0 ± 439.0 (29)	5965.0 ± 290.0 (35)	Sample time	127.3	1	<0.0001
		Post-cold storage	10551.0 ± 1038.0 (23)	14462.0 ± 643.0 (28)	12868.0 ± 915.0 (35)	Spore group	1.91	2	0.151
						Interaction	6.22	1	0.002
						Error		169	
	Lipid concentration (µg /µl)	Pre-cold storage	1217.1 ± 87.6 (23)	1095.3 ± 43.7 (29)	1156.2 ± 49.6 (35)	Sample time	8.20	1	0.005
		Post-cold storage	1027.0 ± 71.6 (23)	1030.4 ± 52.6 (28)	1002.6 ± 48.7 (35)	Spore group	0.49	2	0.613
						Interaction	0.58	1	0.559
						Error		169	
Combined data C-POL and C-SUP	Weight (mg)					Sample time	152.41	1	<0.0001
						Spore group	0.88	2	0.42
						Interaction	1.53	2	0.22
						Error		337	
	Protein concentration (µg/µl)					Sample time	253.0	1	<0.0001
						Spore group	1.29	2	0.28
						Diet	0.07	1	0.80
						Interaction	2.47	2	0.09
						Error		337	
	Lipid concentration (µg/µl)					Sample time	18.1	1	<0.0001
						Spore group	0.58	2	0.56
						Diet	0.21	1	0.65
						Interaction	0.53	2	0.59
						Error		335	

The effects of Fumagillin treatment on fat body metrics were evaluated by comparing control and treated colonies with zero spores per bee. Pre-cold storage fat body weights and lipid concentrations did not differ between F-POL and C-POL, but protein concentrations were lower in F-POL than C-POL (Table 5). Post-cold storage fat body metrics did not differ between C-POL and F-POL. Similar results occurred for C-SUP and F-SUP where pre-cold storage fat body protein concentrations were significantly lower in C-SUP, and weights and lipid concentrations were similar. Post-cold storage fat body metrics did not differ between C-SUP and F-SUP.

**Table 5.** Effects of Fumagillin treatment on fat body metrics in colonies averaging zero spores per bee prior to overwintering in cold storage (pre-cold storage). All fat body measurements were from pooled samples of 10 bees per colony. Colonies were treated with Fumagillin (Fumidil-B) in sugar syrup (treatment) or sugar syrup only (control) 3 wk prior to the hives being put into cold storage for overwintering. Colonies were fed either pollen patty or protein supplement prior to cold storage. *P*-values denoted with an asterisk (*) indicate significant differences between controls and treatments

Diet	Sample time	Fat body metric	Average ± SE		*t*	*P*	*df*
			Control	Treatment			
Pollen	Pre-cold storage	Weight	48.6 ± 1.8	47.5 ± 2.1	0.41	0.68	83
		Protein concentration	6368 ± 505	5160 ± 176	2.26	*0.03	27
		Lipid concentration	1194 ± 68	1090 ± 24	0.82	0.42	27
	Post-cold storage	Weight	79.3 ± 5.4	79.5 ± 3.3	0.04	0.97	40
		Protein concentration	12411 ± 913	12869 ± 578	0.42	0.67	41
		Lipid concentration	1027 ± 61	1011 ± 31	0.24	0.81	34
Protein supplement	Pre-cold storage	Weight	50.0 ± 2.9	47.7 ± 1.0	0.76	0.45	28
		Protein concentration	7008 ± 554	5574 ± 200	2.43	*0.02	29
		Lipid concentration	1217 ± 88	1131 ± 31	0.93	0.36	27
	Post-cold storage	Weight	67.9 ± 6.1	77.9 ± 3.3	1.44	0.16	37
		Protein concentration	10559 ± 993	12614 ± 561	1.80	0.08	37
		Lipid concentration	1027 ± 72	1066 ± 26	0.51	0.61	27

### Should Beekeepers Treat *Nosema*?

All cost estimates for *Nosema* treatments before hives were put into cold storage are based on the recommendation that a 2-story hive (i.e., about 30,000–40,000 adult bees) be treated with 25 g of Fumidil-B added to the sugar syrup and fed to the colony according to label directions (https://dailymed.nlm.nih.gov/dailymed/fda/fdaDrugXsl.cfm?setid=dc684d6c-60f4-4e24-b76a-c07dc0efe35b). Since all colonies would be fed sugar syrup prior to cold storage, only the cost of the Fumidil-B and its application are counted as added expenses. The current price for Fumidil-B treatment per colony is $1.85 (Mann Lake, Hackensack, MN, USA). In year 2 of our study, the cost of treating 176 colonies was $326 ($1.85 * 176). Estimated labor costs for Fumidil-B treatment are based on the additional time required to add and mix the product into the sugar syrup. If Fumidil-B was added to the tank containing sugar syrup to feed 176 hives and thoroughly mixed, we estimate it would take an additional 30 minutes for the treatment. At an hourly wage of $15/h, labor cost for treating 176 colonies would be $7.50 (0.5 h * $15/h). Summing the material and labor costs, a Fumidil-B application before cold storage for 176 colonies would cost $334 ($1.90 per colony).

The average rental price per colony for almond pollination in 2022 was $203 with the lowest rental cost being $165 (https://www.thebeecorp.com/post/2022-almond-pollination-prices). Sixty-three of the 88 F-SUP colonies (71.6%) were large enough to rent for almond pollination compared with 50 out of 88 C-SUP colonies (57%). Gross revenue generated from F-SUP colony rental was $10,395–$12,798 (63 colonies* $165 per colony; 63 colonies * $203 per colony) and net revenue was $10,228–$12,631 ((gross revenue) − ($1.90 per colony * 88 colonies for Fumagillin treatment)). C-SUP colony rental generated $8,250–$10,150. The difference between treated and untreated colonies was $1,636–$1,866.

There was little difference between the number of C-POL and F-POL colonies that were large enough for almond pollination rental. Fifty-four C-POL colonies were large enough to rent and generated a net revenue of $8,910–$10,962 (54 colonies* $165 per colony; 54 colonies * $203 per colony). There were 52 F-POL colonies large enough to rent so gross income from rental fees was $8,580–$10,556 (52 colonies* $165 per colony; 52 colonies * $203 per colony). With the additional cost of the Fumidil-B treatment ($334 for 88 colonies), net revenue for F-POL was $8,246–$10,222 or $664–740 lower than C-POL.

## Discussion

*Nosema* infection levels were measured to determine the relationship between overwintering colony growth and survival. In year 1, colony sizes and the percentage of hives that could be rented for almond pollination after overwintering were not related to infection levels. In an expanded study (year 2), we tested if diet alone and in combination with Fumagillin treatments reduced spore numbers and increased overwintering colony size and survival. Fumagillin treatments significantly lowered spore numbers in colonies fed either pollen or protein supplements compared with untreated controls. However, the percentage of overwintered colonies that could be rented for almond pollination and colony sizes after cold storage or almond bloom were not affected by diet or Fumagillin treatment. Furthermore, Fumagillin may have had negative effects on colony size. Though sizes and survival rates in treated and untreated colonies fed either diet were similar after cold storage, levels of *Nosema* infection were correlated with smaller colonies and lower survival rates. Colonies averaging > 1 × 10^6^ spores per bee were smaller than those averaging zero spores per bee, and fewer could be rented for almond pollination or were alive after almond bloom. *Nosema* did not affect fat body metrics in bees during cold storage. Fumagillin, however, lowered protein concentrations in the fat body and may have contributed to the smaller sizes of treated colonies compared with controls.

Our study might explain some of the inconsistencies in evaluating the impact of *Nosema* on colony losses. Conflicting results might be due to sample sizes and the degree of variability in *Nosema* spore numbers among colonies. In year 1, when we had 39 colonies and too few with zero spores to designate them as a separate group. Post-cold storage colony survival and size did not differ between those with high and low *Nosema* spore loads. However, in year 2, we more than doubled the number of colonies in each treatment group and could separate groups of control colonies based on the presence of spores and their numbers. We detected differences in colony survival and sizes especially after almond bloom, based on average spores per bee. Colonies with > 1 × 10^6^ were smaller, and fewer could be rented for almond pollination than those with lower spore averages. Our results are similar to an earlier study showing that spore number was significantly associated with colony size (Emsen et al. 2020).

Another possible source of inconsistency in evaluating the impact of *Nosema* on colonies might be from when colonies are sampled. *Nosema* infections can have long asymptomatic incubation periods, and measuring the effects during those times could make the infection appear inconsequential (Higes et al. 2007). In our study, colony survival was measured after cold storage and almond bloom. After cold storage, colony survival was similar for both treated and untreated colonies fed either diet and between colonies, averaging either zero or > 1 × 10^6^ spores per bee. However, after almond bloom, highly infected colonies were smaller and had significantly lower survival than those with zero spores per bee. Spore counts can increase over the winter and be high in the spring (Emsen et al. 2020), especially if overwintering bees cannot defecate outside the hive, as occurred in our study. Spores can also be transmitted to young bees through trophallaxis with older infected nestmates or by cleaning comb cells soiled with fecal material during winter confinement. Populations in highly infected colonies may have declined during almond bloom because infected bees have reduced longevity (Eiri et al. 2015, Urbieta-Magro et al. 2019) particularly when levels are ≥ 1 × 10^6^ spores per bee (Emsen et al. 2020). During periods of intense foraging, such as during almond bloom, short-lived bees can die at rates that exceed the emergence of new bees, causing populations to decline. Since brood rearing and adult population size are related, colony death can occur from the combination of short-lived bees, and insufficient brood rearing that creates a downward spiral in population numbers (Betti et al. 2014). In our study, brood rearing was significantly affected by spore number in C-POL colonies. The highly infected colonies died over the 3 - 4 weeks of almond bloom when brood rearing should have been increasing as resources provided by the bloom were plentiful.

The connection between diet and the prevalence of *Nosema* is well documented, but results are inconsistent. In some studies, spore numbers are higher in bees fed protein supplements than pollen patty (Fleming et al. 2015, Jack et al. 2016, DeGrandi-Hoffman et al. 2018, Watkins de Jong et al. 2019, Castelli et al. 2020). The increases in spore numbers might indicate that protein supplements do not provide adequate nutrition to generate strong immune responses. However, adding a small amount of pollen (10%) reduced pathogen levels to those in bees-fed pollen (Watkins deJong et al. 2019). We had similar results where colonies fed a protein supplement with 4% pollen had spore numbers that increased at similar rates between sampling intervals and did not differ from those in colonies fed pollen patties.

Though colonies treated with Fumagillin had lower *Nosema* spore numbers, we might have detected toxic effects from the treatment. F-POL with zero spores prior to cold storage were smaller after cold storage and almond bloom compared with C-POL. F-POL colonies also produced less brood than C-POL and had lower fat-body protein concentrations. Reduction in brood rearing following Fumagillin treatment has been previously reported (Webster 1994). Fumagillin reduces *Nosema* infections because it inhibits methionine aminopeptidase-2 (MetAP2) (Esvaran et al. 2018), an enzyme required for the full activation of many proteins (Lowther and Matthews 2000). The MetAP2 gene in *Nosema* and in other eukaryotes are homologous (Alvarado et al. 2009), so Fumagillin might have affected protein structure and function in bees from treated colonies. Both F-POL and F-SUP with zero spores had lower protein concentrations in their fat bodies than C-POL or C-SUP before cold storage, suggesting effects on protein mobilization shortly after Fumagillin treatment. Lower protein concentrations in treated colonies could have affected adult longevity and brood production (Brodschneider and Crailsheim 2010, Hopkins et al. 2021, DeGrandi-Hoffman et al. 2023) and caused treated colonies to be smaller with less brood than untreated colonies.

Similar to a previous study, fat body metrics changed while colonies were in cold storage (DeGrandi-Hoffman et al. 2023). Weight and protein concentrations increased, and lipid concentrations decreased. Analyses including all colonies in each treatment indicated that pre- and post-cold storage changes in fat body metrics were not affected by *Nosema*. This was surprising since *Nosema* damages midgut tissue and likely impairs nutrient digestion and absorption (Liu 1984, Higes et al. 2007, Dussaubat et al. 2012). *Nosema* also reduces the expression of transcripts related to carbohydrate, protein, and lipid metabolism in infected bees (Holt et al. 2013), so effects on fat body metrics were expected especially for highly infected bees. However, the time of year when bees were infected with *Nosema* might have influenced the results. A study with bees infected with *Nosema* in either spring or fall showed no effect of infection on fat body gene expression in fall bees. Spring bees infected with *Nosema*, however, showed different patterns of expression for numerous genes, including those associated with the TCA cycle and carbon, pyruvate, and lipid metabolism (DeGrandi-Hoffman et al. 2018). Bees examined in our study were collected in the fall and winter, and, similar to earlier studies, they did not show effects of *Nosema* infections on fat body metrics. Results may have been different if we analyzed bees in the spring or summer.

The decision to treat *Nosema* is about weighing the possibilities of greater colony survival if spore numbers are high against the cost and possible undetectable or even negative effects of Fumagillin if spore numbers are low. Our study showed that pre-cold storage colony size and spore numbers were significant predictors of colony suitability for pollination rental. The severity of *Nosema* infections and the risk of losing colonies are difficult to assess for most beekeepers since colonies can be asymptomatic, and a compound microscope with phase-contrast lighting is required to see spores (Fries et al. 2013). Also, as seen in both years of our study, some colonies in an apiary can have no spores, while others have more than a million. Without a clear picture of the possible risk of losing colonies and rental fees to *Nosema* infections, treating for *Nosema* is like buying insurance. If colonies were treated but there was no increase in the number that survived and were rented for pollination, there was no benefit to treating. However, if infection levels are high enough so that colonies would be lost or too small to rent for pollination, then the return on the $1.90 per colony cost for treatment could be substantial.

## Supplementary data

Supplementary data are available at *Journal of Economic Entomology* online.

toae187_suppl_Supplementary_Table_S1

toae187_suppl_Supplementary_Table_S2

## Data Availability

The data will be available at AgCommons under the URL: https://figshare.com/s/1cdcd10de03f0c49cffe.
