# Supplementary material for: Do diet and Fumagillin treatment impact Vairimorpha (Nosema) spp. (Microspora: Nosematidae) infections in honey bees (Hymenoptera: Apidae) and improve survival and growth of colonies overwintered in cold storage?
Source: J Econ Entomol. 2024 Sep 28;117(6):2203–18. doi: 10.1093/jee/toae187 (PMC11682945; doi:10.1093/jee/toae187)
Supplement: toae187_suppl_Supplementary_Table_S1 [file toae187_suppl_supplementary_table_s1.docx]

**Table S1**. Relationships between colony size (combs with adult bees) and average *Nosema* spores per bee before cold storage and colony sizes after cold storage and almond bloom. In year-1, all colonies were fed pollen before overwintering in cold storage. In year-2, colonies were fed either pollen or protein supplement before overwintering in cold storage.

| Year | Response | Predictors | Coefficient | Standard error | d.f. | p | R^2^ |
| --- | --- | --- | --- | --- | --- | --- | --- |
| 1 | Combs with bees - post cold storage | Combs with bees pre-cold storage | 0.77 | 0.19 |  | <  0.0001 |  |
|  |  | Spores per bee | -0.05 | 0.51 |  | 0.92 |  |
|  |  | Error |  |  | 1, 1, 24 |  | 88.1 |
|  | Combs with bees - post almond bloom | Combs with bees pre-cold storage | 0.63 | 0.21 |  |  |  |
|  |  | Spores per bee | 0.33 | 0.56 |  |  |  |
|  |  | Error |  |  | 1, 1, 24 |  | 91.0 |
| 2 (pollen diet) | Combs with bees - post cold storage | Combs with bees pre-cold storage | 0.29 | 0.12 |  | 0.02 |  |
|  |  | Spores per bee | 0.40 | 0.28 |  | 0.16 |  |
|  |  | Error |  |  | 1, 1, 62 |  | 79.0 |
|  | Combs with bees - post almond bloom | Combs with bees pre-cold storage | 0.31 | 0.31 |  | 0.046 |  |
| Year | Response | Predictors | Coefficient | Standard error | d.f. | p | R^2^ |
|  |  | Spores per bee | 0.32 | 0.35 |  | 0.36 |  |
|  |  | error |  |  |  | 1, 1, 62 | 68.8 |
|  |  |  |  |  |  |  |  |
| 2 (protein supplement) | Combs with bees - post cold storage | Combs with bees pre-cold storage | 0.59 | 0.12 |  | <0.0001 |  |
|  |  | Spores per bee | -0.22 | 0.28 |  | 0.44 |  |
|  |  | Error |  |  | 1, 1, 63 |  | 72.5 |
|  | Combs with bees - post almond bloom | Combs with bees pre-cold storage | 0.60 | 0.14 |  | <0.0001 |  |
|  |  | Spores per bee | -0.21 | 0.35 |  | 0.54 |  |
|  |  | Error |  |  | 1, 1, 63 |  | 71.9 |
