# Supplementary material for: Do diet and Fumagillin treatment impact Vairimorpha (Nosema) spp. (Microspora: Nosematidae) infections in honey bees (Hymenoptera: Apidae) and improve survival and growth of colonies overwintered in cold storage?
Source: J Econ Entomol. 2024 Sep 28;117(6):2203–18. doi: 10.1093/jee/toae187 (PMC11682945; doi:10.1093/jee/toae187)
Supplement: toae187_suppl_Supplementary_Table_S2 [file toae187_suppl_supplementary_table_s2.docx]

**Table S2.** Comparisons of fat body dry weights, protein and lipid concentrations in worker honey bees averaging < 1* 10^6^ or > 1 * 10^6^ *Nosema* spores prior to overwintering in cold storage. Measurements were made before (pre-cold storage) and after (post-cold storage) overwintering. Averages are ± standard error. Average weights are the sum of 10 honey bee fat bodies per colony. Sample sizes represent the number of colonies and are shown in parentheses beneath the average.

| Fat body metric | Sample time | Average ± SE  per spore group | | Source | F | d.f. | p |
| --- | --- | --- | --- | --- | --- | --- | --- |
|  |  | < 1 * 10^6^  (n) | > 1 * 10^6^  (n) |  |  |  |  |
| Weight | Pre-cold storage | 51.2 ± 2.1  (16) | 49.5 ± 2.7  (23) | Spore group | 1.43 | 1 | 0.24 |
|  | Post-cold storage | 66.1 ± 5.0  (16) | 59.2 ± 3.8  (23) | Sample time | 11.22 | 1 | 0.001 |
|  |  |  |  | Error |  | 74 |  |
| Protein concentration | Pre-cold storage | 5282 ± 623  (16) | 6486 ± 1084  (23) | Spore group | 1.78 | 1 | 0.19 |
|  | Post-cold storage | 16109 ± 1638  (16) | 11314 ± 1382  (21) | Sample time | 32.61 | 1 | <0.0001 |
|  |  |  |  | Error |  | 73 |  |
| .  Lipid concentration | Pre-cold storage | 1686 ± 91.6 | 1680 ± 89.2 | Spore group | 0.001 | 1 | 0.95 |
|  | Post-cold storage | 860.0 ± 80.5 | 878.8 ± 46.0 | Sample time | 108.92 | 1 | <0.0001 |
|  |  |  |  | Error |  | 73 |  |
